# Data-driven methods for imputing national-level incidence in global burden of disease studies

**DOI:** 10.2471/BLT.14.139972

**Published:** 2015-02-27

**Authors:** Scott A McDonald, Brecht Devleesschauwer, Niko Speybroeck, Niel Hens, Nicolas Praet, Paul R Torgerson, Arie H Havelaar, Felicia Wu, Marlène Tremblay, Ermias W Amene, Dörte Döpfer

**Affiliations:** a Centre for Infectious Disease Control, National Institute for Public Health and the Environment (RIVM), Bilthoven, Netherlands.; b Department of Virology, Parasitology and Immunology, Faculty of Veterinary Medicine, Ghent University, Salisburylaan 133, 9820 Merelbeke, Belgium.; c Institute of Health and Society (IRSS), Université catholique de Louvain, Brussels, Belgium.; d Centre for Statistics, Hasselt University, Diepenbeek, Belgium.; e Department of Biomedical Sciences, Institute of Tropical Medicine, Antwerp, Belgium.; f Section of Veterinary Epidemiology, University of Zürich, Zürich, Switzerland.; g Department of Food Science and Human Nutrition, Michigan State University, East Lansing, United States of America (USA).; h Food Animal Production Medicine Section, School of Veterinary Medicine UW-Madison, Madison, USA.

## Abstract

**Objective:**

To develop transparent and reproducible methods for imputing missing data on disease incidence at national-level for the year 2005.

**Methods:**

We compared several models for imputing missing country-level incidence rates for two foodborne diseases – congenital toxoplasmosis and aflatoxin-related hepatocellular carcinoma. Missing values were assumed to be missing at random. Predictor variables were selected using least absolute shrinkage and selection operator regression. We compared the predictive performance of naive extrapolation approaches and Bayesian random and mixed-effects regression models. Leave-one-out cross-validation was used to evaluate model accuracy.

**Findings:**

The predictive accuracy of the Bayesian mixed-effects models was significantly better than that of the naive extrapolation method for one of the two disease models. However, Bayesian mixed-effects models produced wider prediction intervals for both data sets.

**Conclusion:**

Several approaches are available for imputing missing data at national level. Strengths of a hierarchical regression approach for this type of task are the ability to derive estimates from other similar countries, transparency, computational efficiency and ease of interpretation. The inclusion of informative covariates may improve model performance, but results should be appraised carefully.

## ملخص

**الغرض:**

وضع أساليب تتميز بالشفافية وقابلية التكرار لاحتساب البيانات المفقودة بشأن الإصابة بالمرض على الصعيد الوطني لعام 2005.

**الطريقة:**

قمنا بمقارنة نماذج عديدة لاحتساب معدلات الإصابة بالمرض المفقودة على صعيد البلدان لمرضين من الأمراض المنقولة بالأغذية - داء المقوسات الخلقي والسرطان الكبدي الخلوي ذو الصلة بالأفلاتوكسين. تم افتراض فقدان القيم المفقودة عشوائياً. وتم اختيار متغيرات عوامل التكهن باستخدام أدنى انكماش مطلق وارتداد عامل الانتقاء. وقمنا بمقارنة الأداء التنبؤي لأساليب الاستيفاء البسيط ونماذج الارتداد للتأثيرات المختلطة والعشوائية البايزية. وتم استخدام التحقق المتبادل باستبعاد إحدى القيم لتقدير دقة النموذج.

**النتائج:**

كانت الدقة التنبؤية لنماذج التأثيرات المختلطة البايزية أفضل بشكل كبير عن تلك الخاصة بأسلوب الاستيفاء البسيط في أحد نموذجي المرض. ومع ذلك، نتج عن نماذج التأثيرات المختلطة البايزية فترات تنبؤ أوسع في كلتا فئتي البيانات.

**الاستنتاج:**

تتوفر أساليب عديدة لاحتساب البيانات المفقودة على الصعيد الوطني. وتتمثل نقاط قوة نموذج الارتداد الهرمي لهذا النوع من النهج في قدرته على استخلاص التقديرات من البلدان المشابهة الأخرى والشفافية والكفاءة الحاسوبية وسهولة التفسير. ويمكن أن يحسن إدراج المتغيرات المصاحبة الثرية بالمعلومات من أداء النموذج، غير أنه ينبغي تقييم النتائج بدقة.

## 摘要

**目的:**

开发2005年国家级疾病发病率缺失数据透明和可重复的填补方法。

**方法:**

我们比较填补两种食源性疾病的缺失国家级发病率的几种模型：先天性弓形体病和黄曲霉毒素相关的肝细胞癌。假定缺失值是随机缺失。使用最小一乘收缩和选择算子（LASSO）回归来选择预测变量。我们比较自然推论方法和贝叶斯随机和混合效应回归模型的预测性能。使用留一交叉验证来评估模型的准确性。

**结果:**

在两种疾病模型的其中一种当中，贝叶斯混合效应模型的预测精度明显优于自然推论方法。然而，贝叶斯混合效应模型对两个数据集生成的预测区间都更宽。

**结论:**

国家层面缺失数据有若干种填补方法。此类任务的分层回归方法的优势在于从其他类似国家得出估算值的能力、透明性、计算效率和解释上的方便。增加信息性的协变量可以改善模型性能，但应对其结果进行仔细地评估。

## Résumé

**Objectif:**

Développer des méthodes transparentes et reproductibles pour imputer les données manquantes sur l'incidence d'une maladie au niveau national pour l'année 2005.

**Méthodes:**

Nous avons comparé plusieurs modèles pour imputer les taux d'incidence manquants au niveau national pour deux maladies d'origine alimentaire: la toxoplasmose congénitale et le carcinome hépatocellulaire lié à l'aflatoxine. Les valeurs manquantes sont supposées être manquantes au hasard. Les variables prédictives ont été sélectionnées en utilisant la régression LASSO (*least absolute shrinkage and selection operator*). Nous avons comparé la performance prédictive des approches d'extrapolation naïves et les modèles de régression à effets aléatoires et mixtes bayésiens. La validation croisée «leave-one-out» a été utilisée pour évaluer la précision du modèle.

**Résultats:**

La précision prédictive des modèles des effets mixtes bayésiens était significativement meilleure que celle de la méthode d'extrapolation naïve pour l'un des deux modèles de maladie. Cependant, les modèles d'effets mixtes bayésiens ont produit des intervalles de prédiction plus larges pour les deux ensembles de données.

**Conclusion:**

Plusieurs approches sont disponibles pour imputer les données manquantes au niveau national. Les forces d'une approche de régression hiérarchique de ce type de tâche sont la capacité de dériver des estimations à partir d'autres pays similaires, la transparence, l'efficacité des calculs et la facilité d'interprétation. L'inclusion de covariables informatives peut améliorer la performance du modèle mais les résultats doivent être évalués avec prudence.

## Резюме

**Цель:**

Разработать прозрачные и воспроизводимые методы ввода отсутствующих данных о распространенности заболевания на национальном уровне за 2005 год.

**Методы:**

Производилось сравнение нескольких моделей ввода отсутствующих данных о частоте случаев заболевания на национальном уровне для двух заболеваний пищевого происхождения: врожденный токсоплазмоз и афлатоксин-зависимая гепатоклеточная карцинома. Отсутствующие значения были расценены как не имеющие определенной закономерности. Прогностические переменные были выбраны с использованием наименьшего абсолютного значения естественной убыли и регрессии оператора выбора. Было произведено сравнение прогностической эффективности методов первичной экстраполяции и байесовских регрессионных моделей со случайными и смешанными эффектами. Для оценки точности моделей использовалась перекрестная проверка с исключением.

**Результаты:**

Прогностическая точность байесовских моделей со смешанными эффектами была существенно выше, чем при использовании метода первичной экстраполяции для одной из двух моделей заболевания. Тем не менее, байесовские модели со смешанными эффектами позволили получить более широкие прогностические интервалы для обеих совокупностей данных.

**Вывод:**

Доступно несколько подходов к вводу отсутствующих данных на национальном уровне. Сильными сторонами подхода к решению задач данного типа с использованием метода многоуровневой регрессии являются возможность выведения приблизительных значений на основе данных для других схожих стран, прозрачность, эффективность вычислений и легкость интерпретации. Включение информативных ковариат может повысить эффективность модели, но результаты должны подвергаться тщательной оценке.

## Resumen

**Objetivo:**

Desarrollar métodos transparentes y reproducibles de imputación de datos ausentes sobre la incidencia de la morbilidad a nivel nacional para el año 2005.

**Métodos:**

Se compararon varios modelos de imputación de las tasas ausentes de incidencia a nivel nacional para dos enfermedades transmitidas por los alimentos, la toxoplasmosis congénita y el carcinoma hepatocelular relacionado con la aflatoxina. Se consideró que los valores ausentes faltaban al azar. Las variables de predicción se seleccionaron por medio de la contracción absoluta mínima y la regresión del operador de la selección. Se comparó el rendimiento predictivo de los enfoques de extrapolación ingenua y los modelos bayesianos de regresión al azar y de efectos mixtos, y se empleó una validación cruzada dejando uno fuera para evaluar la exactitud del modelo.

**Resultados:**

La exactitud predictiva de los modelos bayesianos de efectos mixtos fue significativamente mejor que la del método de extrapolación ingenua para uno de los dos modelos de enfermedad. Sin embargo, los modelos bayesianos de efectos mixtos generaron intervalos de predicción más amplios para ambos conjuntos de datos.

**Conclusión:**

Son numerosos los enfoques disponibles para la imputación de datos ausentes a nivel nacional. Los puntos fuertes de un enfoque de regresión jerárquica para este tipo de tareas son la capacidad de obtener estimaciones de otros países similares, la transparencia, la eficiencia informática y la facilidad de interpretación. La inclusión de covariables informativas puede mejorar el rendimiento del modelo, aunque se deben evaluar atentamente los resultados.

## Introduction

An essential prerequisite for estimating global disease burden using summary health metrics, such as the disability-adjusted life year,^1^ is the availability of national-level data on the incidence or prevalence of the disease of interest. For some countries such information is not available, due to financial constraints, lack of surveillance data or other factors.^2^

The World Health Organization (WHO) initiative to estimate the global burden of foodborne diseases, launched in 2006,^3^ is advised by the Foodborne Disease Burden Epidemiology Reference Group.^4^ For applied studies such as this, methods are required to estimate disease burden for countries with missing national-level data. In many studies, extrapolation approaches with little validation have been employed to fill data gaps, e.g. by assigning the value of a certain country or the mean value of neighbouring countries to the country with missing data.^5^^–^^7^ Such methods are arbitrary and do not account for uncertainty arising from the imputation of missing data.

Statistical methods are available for the analysis of incomplete datasets.^8^^–^^10^ Alternatively, there are numerous methods for imputing missing data and for assessing their validity. Given the availability of information for populations both with and without missing data, empirical models are typically fitted to existing data and predictions are generated for missing values from the fitted model. This method, sometimes termed farcasting, by analogy with forecasting, therefore generates predicted health statistics.^11^ An important step in this method is the selection of variables allowing robust predictions.

The imputation methods we investigated require that missingness is uninformative about the missing value, i.e. that missing data are missing at random.^8^ Resource-poor countries may be less likely to report the incidence of a given disease, despite having a higher incidence compared with resource-rich countries. However, provided that the probability of reporting disease incidence is unrelated to disease incidence within the category of resource-poor countries, such data would be considered missing at random. This observation implies that the imputation model should allow for clustering of countries according to resource-richness. Missing data may also be more frequent for less populous nations. However, true disease incidence is rarely related to population size. Imputed incidences for small national populations usually have minor effects on disease burden estimates at regional or global level.

Our goal was to estimate disease incidence and associated uncertainty at national level. We describe a method for comparing and evaluating relatively simple data-driven imputation approaches with data sets employed in global burden of disease studies, assuming that data are missing at random. We present transparent general approaches that can be applied to all countries and multiple diseases.

## Methods

We compared the performance of several imputation approaches using two foodborne diseases – congenital toxoplasmosis and aflatoxin-related hepatocellular carcinoma – for which some published incidence data were available.^12^^,^^13^ Congenital toxoplasmosis, caused by the protozoal parasite *Toxoplasma gondii*, may cause ocular and neurological disorders in the unborn child, possibly leading to stillbirth or neonatal death.^12^ Aflatoxin is a metabolite produced by the fungi *Aspergillus flavus* and *Aspergillus parasiticus* in maize and nuts, and is a known human liver carcinogen.^13^ See Appendix A (available at: http://www.cbra.be/publications/imputation-appendix.pdf) for further details regarding these two example data sets. The goal was to produce a complete set of incidence estimates for WHO Member States for the year 2005.

We compared the performance of various methods using leave-one-out cross-validation, because no external incidence estimates for the missing data were available. This allowed us to estimate the expected predictive accuracy of imputation methods in practice. We classified countries according to 17 food consumption clusters, using the Global Environment Monitoring System’s Food contamination monitoring and assessment programme.^14^ All except 19 countries had been assigned to a food cluster. These 19 countries were assigned to the same cluster as a neighbouring country (Appendix A).

### Imputation approaches

The imputation methods included naive extrapolation and two hierarchical modelling approaches: (i) random effects regression models; and (ii) mixed-effects regression models. The rationale for specifying random effects models is that countries with missing data can “borrow strength’’ from other countries within the same cluster, and clusters with few or no data can ‘‘borrow strength’’ from the global population.

#### Method 1

Foodborne disease incidences among countries with similar food consumption patterns should be similar. Rather than fitting a statistical model to existing data, the naive extrapolation approach imputes missing incidences as the median of all other countries with data within the same food cluster. If data were missing for all countries within a cluster, the global median was assigned. Country-specific 95% prediction intervals were derived via bootstrapping with replacement. A thousand bootstrap samples of the number of countries with observed data were taken and an identical method applied to each sample. The unit of sampling was the country-level incidence.

#### Method 2

This Bayesian random effects regression model applies a single random effect serving as a clustering variable for different countries. We explored two possible clustering variables: food cluster (*n* = 17) and WHO subregion (*n* = 14). To normalize the distribution of the outcome variable, the observed incidences were log-transformed, leading to a log-normal regression model. Other approaches to deal with skewed data were considered such as a normal model with log-link or a gamma model, but none were found to differ meaningfully from the log-normal model. Equations for this model can be found in Appendix A.

Vague normal priors were specified for the random model coefficients and for hyper-parameters. Posterior distributions were derived through Markov chain Monte Carlo methods using the rjags package^15^^,^^16^ in R software version 3.0.2 (R Foundation for Statistical Computing, Vienna, Austria). Two chains were initiated of 20 000 posterior samples each, with the first 15 000 discarded. Standard graphical indicators of non-convergence were checked. Predicted values were taken from the posterior predictive distribution. Example code is provided in Appendix A.

#### Method 3

This Bayesian mixed-effects regression model extends Method 2 by including disease-specific covariates, leading to a mixed-effects model. Again, the model was applied to the log-transformed observed incidences, and either food cluster or WHO subregion was included as a random effect. Method 3 relies on predictor variables measured at the national level. Our hypothesis is that covariates capturing the socioeconomic, food production-related and the public health and hygiene situation within a given country will be informative of between-country variation in the incidence of foodborne diseases. We describe below how a common set of potential covariates was derived from publicly available databases.

World development indicators,^17^ the data repository of world health statistics^18^ and the database of the Food and Agriculture Organization of the United Nations (FAOstat)^19^ were the starting point for defining an initial set of 1200 covariates. This was reduced to 194 variables related to food- or waterborne disease, food production and consumption, agriculture, environment, health, demography, economics and development. If the data point for the year 2005 was missing, subsequent years were searched (2006 to 2011 for world development indicators and world health statistics variables and 2006 to 2009 for FAOstat variables), and the nearest year with non-missing data was used. If two variables were highly correlated, one was removed, with the retained member chosen according to relevance as a general indicator (for instance, total mortality rate was chosen in preference to either male-only or female-only mortality rate). To the remaining 112 variables, an arbitrary missingness threshold of 26% was applied to limit the number of missing values, reducing the data set to 65 variables. A final set of 51 variables resulted when non-numeric variables were removed (Appendix A). Any missing values in these remaining 51 variables were imputed using the mice package for R.^20^ One hundred imputed data sets were generated using the predictive mean matching method. This is useful for bounded variables such as proportions, since imputed values are sampled only from observed values. The simple mean of each set of 100 imputed data sets was calculated, implying that uncertainty in imputed values was ignored. Nine covariates were log-transformed following inspection of normal quantile-quantile plots.

The use of principal components analysis was explored to further reduce the set of 51 covariates. However, as this step did not increase model performance, it was excluded from the reported results.

### Analysis

Fixed effects were selected from the set of potential covariates (Appendix A) in a data-driven, stepwise manner. First, least absolute shrinkage and selection operator regression was used to select covariates with non-zero regression coefficients.^21^ We optimized the covariate estimates and model fit using the tuning parameter lambda.^22^^,^^23^ Least absolute shrinkage and selection operator models were fitted using the glmnet package for R.^24^^,^^25^

Subsequently, backward-stepwise elimination based on Akaike's information criterion further reduced the covariate set resulting from the least absolute shrinkage and selection operator step, yielding a subset of covariates that were significantly associated with disease incidence.^23^^,^^25^

Model implementation was the same as for the random effects model (Method 2). As before, vague normal priors were specified for the model coefficients and hyper-parameters. Example JAGS code is provided in Appendix A.

### Predictive accuracy

The accuracy of the model predictions was evaluated using leave-one-out cross-validation.^26^ Data for a single country were temporarily deleted and the remaining data used in an attempt to recover this deleted value. The procedure was repeated by holding out and predicting each country in turn. The mean absolute prediction error was computed as the prediction error averaged over all held-out countries. Ninety-five percent confidence intervals (CI) around mean absolute prediction error values were computed via bootstrapping methods; 10 000 samples with replacement were used, and the mean absolute prediction error was calculated for each sample separately.

To compare the predictive performance of the three models, we applied the Wilcoxon signed-ranks test to the paired absolute prediction errors obtained from each imputation method. We used Bonferroni's correction for multiple comparisons.

### Effect of database size

We estimated the effect of varying the number of observations in the database on the central estimate and 95% CI of the mean absolute prediction error. As an example, we used a mixed-effects regression model of toxoplasmosis. To simplify the analysis, a frequentist version was used, yielding virtually identical results. The database size was reduced from 115 to 15 countries in steps of five. Mean absolute prediction error values for each reduced database size and bootstrapped 95% CI were computed by taking 100 random samples of the specified size from the set of countries with data, and calculating mean absolute prediction error separately for each sample.

## Results

### Congenital toxoplasmosis

We obtained the toxoplasmosis incidence per 1000 live births for 118 countries, implying 74 countries without data. Table 1 shows the mean absolute prediction error for each method and variant. We compared the predictive accuracy between the three methods. For Method 2 and Method 3 we also assessed the effect of including each of the two possible clustering variables as random effect. Compared with Method 1, predictive accuracy was greater for both Method 3 variants (*P* < 0.05), while both Method 2 variants were statistically indistinguishable. In general, Method 3 generated larger prediction intervals (Table 1 and Appendix A). Eight covariates were retained for Method 3, based on an Akaike's information criterion penalty threshold of six. These were: percent arable land, percent urban population, annual precipitation, CO_2_ emissions, rice supply, agricultural value (as percentage of total gross domestic product), neonatal mortality rate and fresh-water sources.

**Table 1:** Comparison of three methods for imputing missing incidence data for congenital toxoplasmosis, 2005

Method	Mean absolute prediction error (95% CI)	Global incidence per 1000 live births (95% prediction interval)
**Method 1: median of other countries within same cluster^a^**	0.65 (0.55–0.75)	1.47 (1.45–1.49)
**Method 2: Bayesian random effects regression**		
Food cluster random effect	0.62 (0.54–0.72)	1.44 (1.38–1.58)
WHO subregion random effect	0.55 (0.47–0.63)	1.45 (1.39–1.61)
**Method 3: Bayesian mixed effects regression^b^**		
Food cluster random effect	0.54 (0.47–0.61)	1.50 (1.42–1.72)
WHO subregion random effect	0.53 (0.46–0.60)	1.52 (1.42–1.76)

CI: confidence interval; WHO: World Health Organization.

^a^ Countries were clustered based on food consumption using the global environment monitoring system – food contamination monitoring and assessment programme.^14^

^b^ The covariate set consisted of percent arable land, percent urban population, annual precipitation, CO_2_ emissions, rice supply, agricultural value added, neonatal mortality rate and fresh-water sources.

**Fig. 1:**
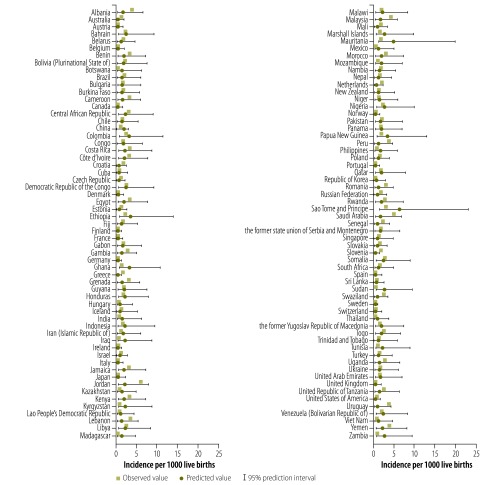
Comparison of observed and predicted incidence rate of congenital toxoplasmosis in 118 countries, 2005

Note: Imputed values were derived from Bayesian mixed effects regression with WHO subregion as random effect.

**Fig. 2:**
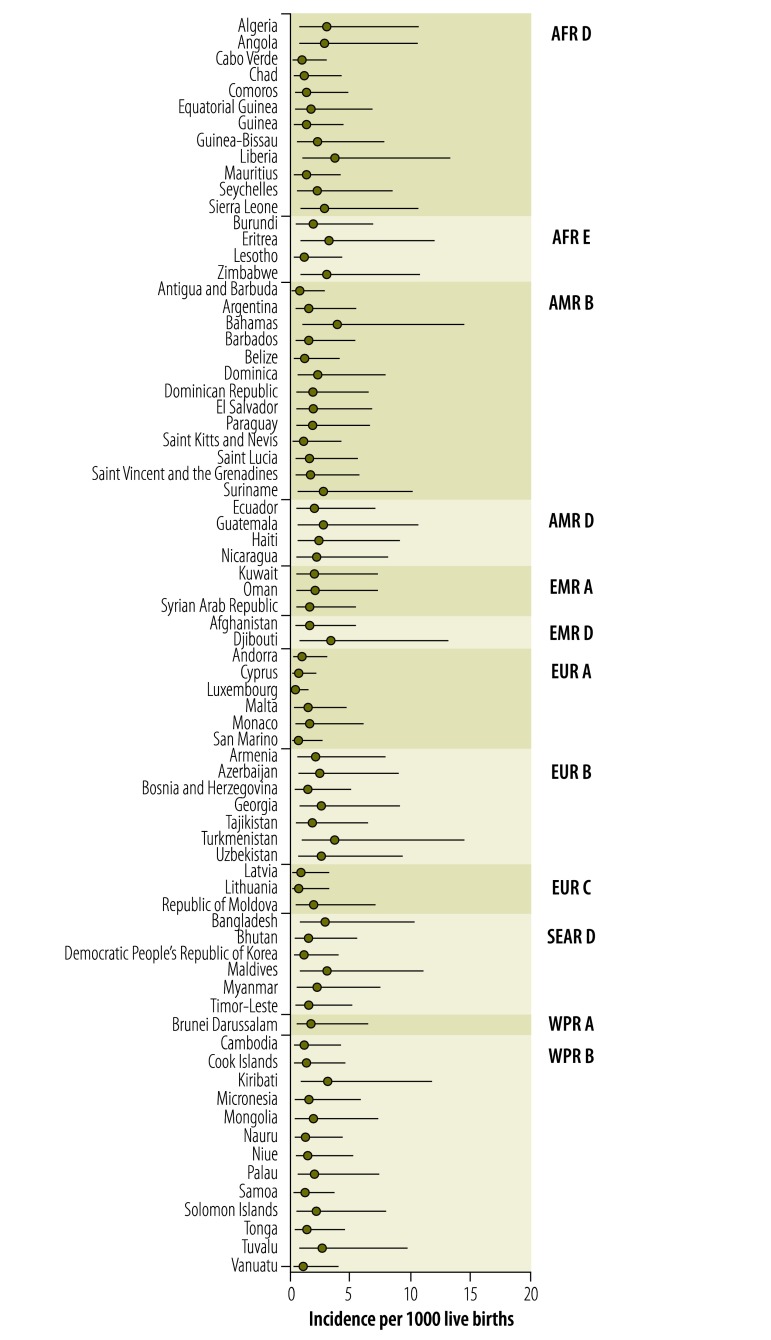
Predicted incidence rate of congenital toxoplasmosis for 74 countries with missing data, 2005

AFR: African Region; AMR: Region of the Americas; EMR: Eastern Mediterranean Region; EUR: European Region; SEAR: South-East Asia Region; WPR: Western Pacific Region.

Note: Bayesian mixed effects regression with WHO subregion as random effect. Countries are ordered according to WHO subregion (http://www.who.int/choice/demography/regions). Error bars represent 95% prediction intervals.

Fig. 1 compares original and imputed incidence values, derived using Method 3, with WHO subregion as random effect, for the 118 countries with non-missing data. Imputed incidence values for the 74 countries with missing data, also derived using Method 3, are displayed in Fig. 2. A comparison of the three methods in terms of the global incidence per 1000 live births is provided in Table 1 and by WHO subregion in Appendix A.

### Aflatoxin-related hepatocellular carcinoma

We obtained the incidence of hepatocellular carcinoma per 100 000 population for 33 countries, implying 159 countries without data. Table 2 shows the mean absolute prediction error for each method. The performances of both Method 2 and Method 3 variants were not statistically distinguishable from that of Method 1. As seen for toxoplasmosis, Method 3 generated larger prediction intervals (Table 2 and Appendix A). Three covariates were retained for Method 3, i.e. food supply from animal products, percentage of population subject to tuberculosis infection and energy use (defined as kg of oil equivalent per capita).

**Table 2:** Comparison of three methods for imputing missing incidence data for aflatoxin-related hepatocellular carcinoma, 2005

Method	Mean absolute prediction error (95% CI)	Global incidence per 100 000 population (95% prediction interval)
**Method 1: median of other countries within same cluster^a^**	1.27 (0.93–1.64)	1.13 (1.10–1.16)
**Method 2: Bayesian random effects regression**		
Food cluster random effect	1.24 (0.97–1.54)	1.00 (0.91–1.44)
WHO subregion random effect	1.23 (0.95–1.55)	1.05 (0.91–1.64)
**Method 3: Bayesian mixed effects regression^b^**		
Food cluster random effect	1.08 (0.87–1.31)	1.17 (0.94–3.77)
WHO subregion random effect	1.08 (0.85–1.32)	1.19 (0.94–3.50)

CI: confidence interval; WHO: World Health Organization.

^a^ Countries were clustered based on food consumption using the global environment monitoring system – food contamination monitoring and assessment programme.^14^

^b^ The covariate set consisted of food supply from animal products, percentage of population subject to tuberculosis infection and energy use.

**Fig. 3:**
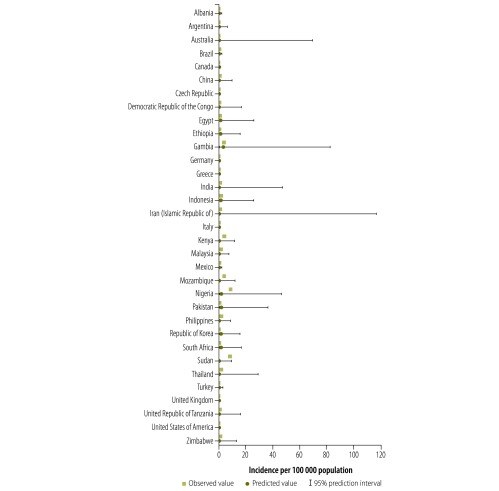
Comparison of observed and predicted incidence rate of aflatoxin-related hepatocellular carcinoma in 33 countries, 2005

Note: Imputed values were derived from Bayesian mixed effects regression with WHO subregion as random effect.

Fig. 3 shows original and imputed incidence values derived using the method with the lowest mean absolute prediction error – i.e. Method 3 with WHO subregion as random effect – for the 33 countries with non-missing data.

### Effect of database size

**Fig. 4:**
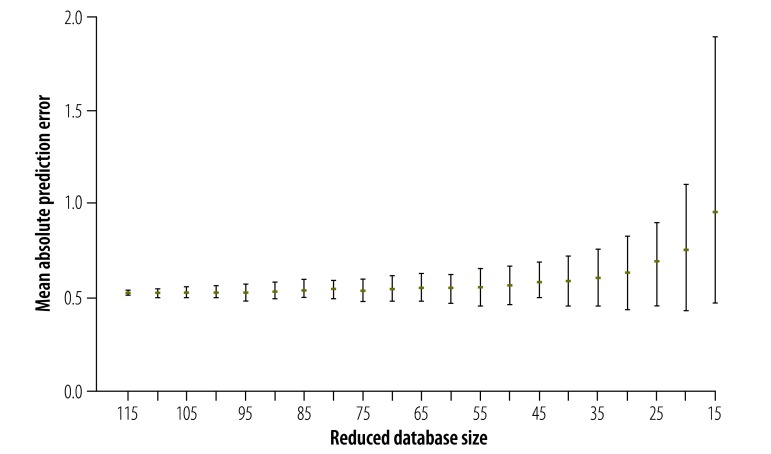
Expected mean absolute prediction error associated with database size for congenital toxoplasmosis

Note: Bayesian mixed effects regression with WHO subregion as random effect. Error bars represent bootstrapped 95% confidence intervals.

We used Method 3 to impute missing values for toxoplasmosis after deleting some observations from the database. With decreasing database size, both the mean and the variability of the mean absolute prediction error tended to increase (Fig. 4).

## Discussion

We compared the performance of a simple extrapolation method to imputation approaches using regression models. Variation in predictive accuracy across regression-based methods was small. For only one of the two datasets, the Bayesian mixed-effects regression models performed significantly better than the baseline Method 1.

The data-driven approach to selection of covariates for the mixed-effects model retained eight covariates for toxoplasmosis and three for hepatocellular carcinoma. For toxoplasmosis, these included percent urban population and neonatal mortality rate, which are proxy variables for socioeconomic development and general population health. The retained covariates for hepatocellular carcinoma included food supply from animal products and energy use. Transmission of foodborne infection or the risk of contamination may be associated with non-health related variables which serve as proxies for variations in public health and hygiene between countries.

To quantitatively evaluate the performance of any prediction method, validation is necessary.^11^ For our example diseases, internal validation was the only feasible choice, because no external data sources were available. However, selection of imputation approaches should not be based exclusively on numerical criteria such as prediction error, but also on biological plausibility, computational considerations, measures of uncertainty and user-friendliness. Such criteria become even more relevant when dealing with sparse data sets. Imputed values should be reported with an assessment of their uncertainty and interpreted by at least one disease expert.

It seems intuitive to include biologically plausible covariates in the model, either selected through a data-driven approach or directly provided by expert opinion. Our results show that inclusion of disease-specific covariates may be associated with greater prediction error but increased predictive performance compared to naive Method 1. However, as evaluations were only based on internal validation, these results should be interpreted with caution. Indeed, better predictive performance may result merely from overfitting of available data, especially with sparse data sets. Also, out-of-range predictions become more likely with decreasing dataset size. As a result, the inclusion of covariates can lead to unexpected results and should be appraised carefully by disease experts.

A high proportion of missing data might limit the application of these methods. Deleting observations from the toxoplasmosis database demonstrated the degree of bias and variability that may occur when applying imputation methods to small numbers of existing data points. This issue needs to be considered when presenting and interpreting health statistics that draw upon model-based predictions.

We applied the imputation techniques to published point estimates of disease incidence, although estimates of uncertainty were also provided with the original data sets. To fully represent the uncertainty in predictions, Monte Carlo sampling could be applied to take into account uncertainty in national-level incidence. However, for purposes of comparing the degree of bias across imputation methods, the point estimate was considered adequate.

The quality of any imputation model depends on the quality of available data and underlying study designs. In the current case, imputed incidences and estimated uncertainty depend on existing national-level data, while for the mixed-effects model, appropriate data on covariates were also required. If any of these indicators are unreliable, or have a non-monotonic relationship with disease incidence, then predictive abilities of models would be compromised.^11^ The degree of bias introduced by the preliminary multiple imputation step to fill in missing covariate values is not known.

If missingness and disease incidence are associated, the missing at random assumption is invalid. However, establishing such an association is difficult, since the necessary incidence data are not available (Appendix A). We were unable to estimate the degree of potential bias resulting from fitting models to disease incidence data from resource-rich countries to predict incidence rates for countries with fewer resources.

We have restricted our analysis to Bayesian methods. However, if uninformative priors are specified, Bayesian and frequentist statistical frameworks generate similar results. The advantage of Bayesian methods is that the within- and between-region variability can be modelled separately. Furthermore, informative priors for regression coefficients could be specified based on previous analyses of other foodborne diseases with similar incidence distributions. This can be particularly relevant for sparse data sets, with the caveat that in such settings the priors can strongly influence the results.

Selection of covariates required several assumptions, and thresholds for selection were not guided by evidence alone. Ideally, imputations should be done in a context-specific manner, with disease experts introducing specific covariates for which sufficient global data are available or eliminating certain possibilities depending, for instance, on food consumption habits. Although time-consuming, this would improve the biological plausibility of the models.

Finally, the least absolute shrinkage and selection operator method is known for instability with respect to the set of non-zero covariates retained. Slight changes in the data can result in very different sets of retained covariates.^27^ Ideally, covariate sets would be selected during each cross-validation run separately; however, this was impractical. We had to assume that the data are independent. Least absolute shrinkage and selection operator penalized regression methods ignore dependency, although recent developments try to accommodate for this. Nevertheless, least absolute shrinkage and selection operator methods are relatively robust to collinearity of covariates.^25^

## Conclusion

Imputation will never be a perfect substitute for actual data.^28^^,^^29^ We evaluated the predictive accuracy of various methods to impute missing national-level disease parameters. We described data-driven methods for reducing a large data set of socioeconomic, food production, and demographic indicator variables, which may be used to complement imputation models. Hierarchical models, specifying structural relationships between countries, can be a useful approach to the problem of estimating missing national incidence.

## References

[1] Murray CJ, Lopez AD. Measuring the global burden of disease. N Engl J Med. 2013 8 1;369(5):448–57. 10.1056/NEJMra1201534 23902484

[2] Leach-Kemon K, Lopez AD, Lozano R, Naghavi M, Vos T, Speyer P, et al. Filling gaps in all-cause and cause-specific mortality and disability data are essential for improving Global Burden of Disease estimation: descriptive study of missing data by country and region. Lancet. 2013;381:S82 10.1016/S0140-6736(13)61336-7

[3] Kuchenmüller T, Hird S, Stein C, Kramarz P, Nanda A, Havelaar AH. Estimating the global burden of foodborne diseases–a collaborative effort. Euro Surveill. 2009 5 7;14(18):19195. 19422776 10.2807/ese.14.18.19195-en

[4] Havelaar AH, Cawthorne A, Angulo F, Bellinger D, Corrigan T, Cravioto A, et al. WHO initiative to estimate the global burden of foodborne diseases. Lancet. 2013;381:S59 10.1016/S0140-6736(13)61313-6

[5] Resnikoff S, Pascolini D, Etya’ale D, Kocur I, Pararajasegaram R, Pokharel GP, et al. Global data on visual impairment in the year 2002. Bull World Health Organ. 2004 11;82(11):844–51. 15640920 PMC2623053

[6] Gustavsson A, Svensson M, Jacobi F, Allgulander C, Alonso J, Beghi E, et al.; CDBE2010Study Group. Cost of disorders of the brain in Europe 2010. Eur Neuropsychopharmacol. 2011 10;21(10):718–79. 10.1016/j.euroneuro.2011.08.008 21924589

[7] Pullan RL, Smith JL, Jasrasaria R, Brooker SJ. Global numbers of infection and disease burden of soil transmitted helminth infections in 2010. Parasit Vectors. 2014;7(1):37. 10.1186/1756-3305-7-37 24447578 PMC3905661

[8] Rubin DB. Inference and missing data. Biometrika. 1976;63(3):581–92. 10.1093/biomet/63.3.581

[9] Gelman A, Hill J. Data analysis using regression and multilevel/hierarchical models. Cambridge: Cambridge University Press; 2007.

[10] Schafer JL. Analysis of incomplete multivariate data. Boca Raton: CRC press; 1997 10.1201/9781439821862

[11] Murray CJ. Towards good practice for health statistics: lessons from the Millennium Development Goal health indicators. Lancet. 2007 3 10;369(9564):862–73. 10.1016/S0140-6736(07)60415-2 17350457 PMC7137868

[12] Torgerson PR, Mastroiacovo P. The global burden of congenital toxoplasmosis: a systematic review. Bull World Health Organ. 2013 7 1;91(7):501–8. 10.2471/BLT.12.111732 23825877 PMC3699792

[13] Liu Y, Wu F. Global burden of aflatoxin-induced hepatocellular carcinoma: a risk assessment. Environ Health Perspect. 2010 6;118(6):818–24. 10.1289/ehp.0901388 20172840 PMC2898859

[14] Sy MM, Feinberg M, Verger P, Barré T, Clémençon S, Crépet A. New approach for the assessment of cluster diets. Food Chem Toxicol. 2013 2;52:180–7. 10.1016/j.fct.2012.11.005 23182740

[15] Plummer M. JAGS: A program for analysis of Bayesian graphical models using Gibbs sampling. In: Hornik K, Leisch F, Zeileis A, editors. Proceedings of the 3rd International Workshop on Distributed Statistical Computing (DSC 2003); 2003 Mar 20–22, Vienna, Austria. Vienna: R Foundation for Statistical Computing; 2003. pp. 20–2. Available from: http://www.ci.tuwien.ac.at/Conferences/DSC-2003/Proceedings/ [cited 2015 Feb 19].

[16] Plummer M. rjags: Bayesian graphical models using MCMC. R package version 3–10. Vienna: R Foundation for Statistical Computing; 2013. Available from: http://cran.r-project.org/web/packages/rjags/index.html [cited 2015 Feb 21].

[17] World development indicators 2013. Washington: World Bank Publications; 2013.

[18] Global health observatory [Internet]. Geneva: World Health Organization; 2014. Available from: http://www.who.int/gho/en/ [cited 2014 April 13].

[19] FAOstat [Internet]. Rome: Food and Agriculture Organization of the United Nations; 2015. Available from: http://faostat3.fao.org/home/E/ [cited 2015 Feb 21].

[20] van Buuren S, Groothuis-Oudshoorn K. MICE: Multivariate imputation by chained equations in R J Stat Softw. 2011;45:1–67.

[21] Tibshirani R. Regression shrinkage and selection via the lasso. J R Stat Soc Series B Stat Methodol. 1996;58:267–88.

[22] Friedman J, Hastie T, Tibshirani R. Regularization paths for generalized linear models via coordinate descent. J Stat Softw. 2010;33(1):1–22. 20808728 PMC2929880

[23] Hastie T, Tibshirani R, Friedman J. The elements of statistical learning. New York: Springer; 2009 10.1007/978-0-387-84858-7

[24] Friedman J, Hastie T, Tibshirani R. glmnet: Lasso and elastic-net regularized generalized linear models. R package version 1.9–5. Vienna: R Foundation for Statistical Computing; 2009. Available from: http://cran.r-project.org/web/packages/glmnet/index.html [cited 2015 Feb 21].

[25] James G, Witten D, Hastie T, Tibshirani R. An introduction to statistical learning. New York: Springer; 2013 10.1007/978-1-4614-7138-7

[26] Kuhn M, Johnson K. Applied predictive modeling. New York: Springer; 2013 10.1007/978-1-4614-6849-3

[27] Zhao P, Yu B. On model selection consistency of Lasso. J Mach Learn Res. 2006;7:2541–63.

[28] Devleesschauwer B, Ale A, Duchateau L, Dorny P, Lake R, Dhakal P, et al. Understanding the burden of disease in Nepal: a call for local evidence. J Nepal Health Res Counc. 2013 5;11(24):221–4. 24362617

[29] Devleesschauwer B, Ale A, Torgerson P, Praet N, Maertens de Noordhout C, Pandey BD, et al. The burden of parasitic zoonoses in Nepal: a systematic review. PLoS Negl Trop Dis. 2014;8(1):e2634. 10.1371/journal.pntd.0002634 24392178 PMC3879239

